# Influenza A Virus as a Predisposing Factor for Cryptococcosis

**DOI:** 10.3389/fcimb.2017.00419

**Published:** 2017-09-26

**Authors:** Lorena V. N. Oliveira, Marliete C. Costa, Thaís F. F. Magalhães, Rafael W. Bastos, Patrícia C. Santos, Hellem C. S. Carneiro, Noelly Q. Ribeiro, Gabriella F. Ferreira, Lucas S. Ribeiro, Ana P. F. Gonçalves, Caio T. Fagundes, Marcelo A. Pascoal-Xavier, Julianne T. Djordjevic, Tania C. Sorrell, Daniele G. Souza, Alexandre M. V. Machado, Daniel A. Santos

**Affiliations:** ^1^Laboratório de Micologia, Departamento de Microbiologia, Instituto de Ciências Biológicas, Federal University of Minas Gerais, Belo Horizonte, Brazil; ^2^Laboratório de Interação Micro-organismo Hospedeiro, Departamento de Microbiologia, Instituto de Ciências Biológicas, Federal University of Minas Gerais, Belo Horizonte, Brazil; ^3^Departamento de Farmácia, Universidade Federal de Juiz de Fora–Campus Governador Valadares, Centro, Governador Valadares, Brazil; ^4^Centro de Pesquisas René Rachou (CPqRR)/Fundação Oswaldo Cruz (Fiocruz Minas), Belo Horizonte, Brazil; ^5^Centro de Pesquisa e Desenvolvimento de Fármacos, Instituto de Ciências Biológicas, Federal University of Minas Gerais, Belo Horizonte, Brazil; ^6^Marie Bashir Institute for Infectious Diseases and Biosecurity, University of Sydney and Westmead Institute for Medical Research, Westmead, NSW, Australia

**Keywords:** Cryptococcosis, *Cryptococcus gattii*, influenza A H1N1, co-infection, risk factor

## Abstract

Influenza A virus (IAV) infects millions of people annually and predisposes to secondary bacterial infections. Inhalation of fungi within the *Cryptococcus* complex causes pulmonary disease with secondary meningo-encephalitis. Underlying pulmonary disease is a strong risk factor for development of *C. gattii* cryptococcosis though the effect of concurrent infection with IAV has not been studied. We developed an *in vivo* model of Influenza A H1N1 and *C. gattii* co-infection. Co-infection resulted in a major increase in morbidity and mortality, with severe lung damage and a high brain fungal burden when mice were infected in the acute phase of influenza multiplication. Furthermore, IAV alters the host response to *C. gattii*, leading to recruitment of significantly more neutrophils and macrophages into the lungs. Moreover, IAV induced the production of type 1 interferons (IFN-α4/β) and the levels of IFN-γ were significantly reduced, which can be associated with impairment of the immune response to *Cryptococcus* during co-infection. Phagocytosis, killing of cryptococci and production of reactive oxygen species (ROS) by IAV-infected macrophages were reduced, independent of previous IFN-γ stimulation, leading to increased proliferation of the fungus within macrophages. In conclusion, IAV infection is a predisposing factor for severe disease and adverse outcomes in mice co-infected with *C. gattii*.

## Introduction

Influenza A virus (IAV) is a negative stranded RNA virus, which belongs to the *Orthomyxoviridae* family. Seasonal influenza causes 3–5 million cases of severe illness and about 500,000 deaths annually (Krammer and Palese, 2015). It is well-recognized that severe influenza infections predispose to secondary bacterial pneumonia, e.g., caused by *Streptococcus* spp. and *Staphylococcus* spp. (Peltola and McCullers, 2004; Tanaka et al., 2013; Duvigneau et al., 2016). In addition, co-infection with IAV is well-described for the fungus *Aspergillus* spp. (Kwon et al., 2013; Alshabani et al., 2015; Crum-Cianflone, 2016; Nulens et al., 2017); however there are few studies of co-infection with other important respiratory fungal pathogens, e.g., *Cryptococcus* spp. The consequences of the co-infection with IAV-cryptococci are poorly studied, being only available in two case reports describing the secondary cryptococcal meningitis during the 2009-pandemic influenza A H1N1 (Hosseinnezhad and Rapose, 2012; Gupta et al., 2015).

Cryptococcosis is an emerging fungal disease caused by *Cryptococcus neoformans* and *C. gattii* that initially affects lungs, followed by dissemination to the central nervous system (CNS), causing severe meningoencephalitis (Chen et al., 2012, 2014; May et al., 2016). *C. neoformans* affects mainly immunocompromised patients and *C. gattii*, immunocompetent individuals (Kronstad et al., 2011; Bielska and May, 2016). It is estimated 1 million people per year develop cryptococcosis, with about 650,000 deaths (Park et al., 2009; Almeida et al., 2015).

Based on case-control studies in hospitalized patients, the main predisposing factors for *C. gattii* and *C. neoformans* infections are HIV, organ transplantation, decompensated hepatic cirrhosis, cell-mediated immune suppression, autoimmune diseases, pneumonia, other pulmonary disorders, and invasive cancer (Lin et al., 2015). Such studies excluded patients with self-limiting infectious diseases such as viral and easily treated bacterial infections (MacDougall et al., 2011; Pappas, 2013). Notably, according to Pappas (2013), 17–22% of non-HIV and non-transplant patients are considered healthy hosts. These patients manifest more severe complications and permanent neurological sequelae than those with underlying immunocompromise (Pappas, 2013).

Influenza occurs worldwide and *C. gattii* is an emergent and potentially epidemic respiratory fungus, which can cause chronic and severe disease. Moreover, the clinical evidence of co-infection for these pathogens has been reported in humans (Hosseinnezhad and Rapose, 2012; Gupta et al., 2015). In this context, Hosseinnezhad and Rapose (2012) suggested that prolonged influenza A (H1N1) virus infection causes immunologic defects and acute respiratory distress syndrome, which may represent a further emerging risk factor for the development of cryptococcosis in previously healthy individuals. For this reason, we propose that co-infection with influenza and *C. gattii* worsens disease severity and outcomes and here report our investigation of this hypothesis using a murine model.

## Materials and methods

### Cell lines and micro-organisms

Madin-Darby Canine Kidney (MDCK) cells were maintained at 37°C and 5% CO_2_ in complete Dulbecco's modified Eagle Medium (DMEM; SIGMA). Virus stocks of the mouse-adapted influenza A/PR8/34 (H1N1) virus were prepared and titrated on MDCK cells as previously described (Barbosa et al., 2014). *C. gattii* (Cg) VGII, strain L27/01 (UFMG-CM-Y6141) was cultured on Sabouraud's Dextrose Agar (SDA) by 48 h at 37°C (Santos et al., 2014). Other *Cryptococcus* strains, such as, *C. gattii* R265 (VGII) and *C. neoformans* H99 (VNI) were also used for mice survival analysis.

### Mice and ethics statement

Female C57BL/6 mice (6–8 weeks old) were obtained from the animal facilities of the Universidade Federal de Minas Gerais. All experimental procedures were carried out according to the standards of the Brazilian Society of Laboratory Animal Science/Brazilian College for Animal Experimentation (available at www.sbcal.org.br) and Brazilian Federal Law 11,794. The animal studies were approved by the Ethical Commission on Animals Use of the Universidade Federal de Minas Gerais (CEUA/UFMG, protocol n° 354/2015).

### Infection and co-infection experiments

**Figure 1:**
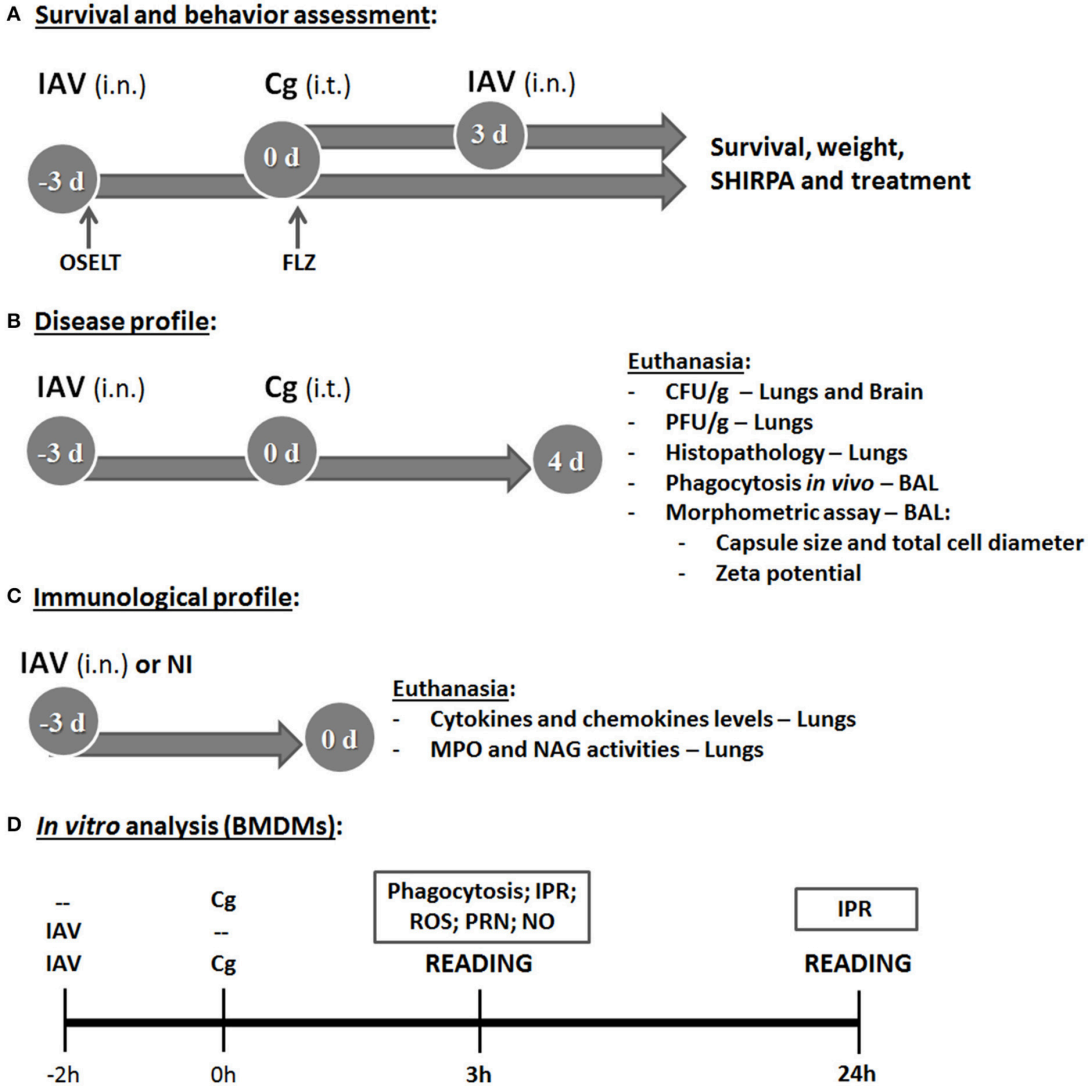
Study design. The first aim was to determine the survival and behavior alterations (using the SHIRPA protocol) in mice co-infected with influenza A virus (IAV) and *C. gattii* **(A)**. The top arrow indicates group of mice (C57BL/6) infected with 1 × 10^4^ CFU of *C. gattii* (Cg) intratracheally (i.t.) at day 0 (0 d), followed by intranasally (i.n.) infection with 1 × 10^3^ PFU of influenza A virus (IAV) after 3 days of Cg infection. The bottom arrow indicates the group infected i.n. with 1 × 10^3^ PFU of IAV 3 days before infection (−3 d: −3 d.b.i) with 1 × 10^4^ CFU of Cg i.t. (day 0). The control groups were mice infected only with Cg, only with IAV and non-infected. The survival, weight and behavior alterations of these animals were monitored daily. After that, the co-infected group of IAV 3 d.b.i. and Cg was chosen for the next experiments. The same strategy of co-infection was used to determine the survival during the treatment of mice with the antiviral Oseltamivir, 31 mg/kg/day (OSELT) or antifungal fluconazole, 10 mg/kg/day (FLZ); each drug was administered intraperitoneally daily and started at the day of infection with IAV (OSELT) or Cg (FLZ). The second aim was to evaluate the disease profile **(B)**. Mice were co-infected with IAV 3 d.b.i. and with Cg (IAV+Cg) or infected only by Cg; IAV or non-infected (NI). Four days post-infection (4 d) with Cg (equivalent to 7 days infection with IAV), these animals were ethically euthanized to assess fungal (CFU/g) and viral burden (PFU/g) in the organs (lungs and brain) and histopathological analysis; also the bronchoalveolar lavage fluid (BAL) was aspired to determine *in vivo* phagocytosis rate and the morphometric analysis of colonies recovered from BAL. Further, our aim was to verify the role of influenza in immunological profile of mice at the time of Cg infection **(C)**. Mice were infected only with IAV (1 × 10^3^ PFU) or NI and were ethically euthanized after 3 days, then cytokines and chemokine levels were measured in the lungs. Also, the myeloperoxidase (MPO) and N-acetylglucosamidase (NAG) activities were quantified as an indirect measurement of neutrophil and macrophage accumulation in lungs, respectively. Lastly, the *in vitro* analysis using bone marrow derived macrophages (BMDM) **(D)** was performed to assess the influence of IAV infection on Cg phagocytosis and intracellular proliferation rate (IPR), as well as measuring the production of reactive oxigen species (ROS), peroxynitrite (PRN), and nitric oxide (NO). BMDM were infected with IAV 2 h before Cg infection and the reading was performed after 3 or 24 h of BMDM Cg infection. Control groups were BMDM infected with only IAV or only Cg. All the experiments were performed at least twice to confirm the data and the results were always reproducible.

Six female C57BL/6 mice per group were anesthetized by intraperitoneal injection of ketamine (60 mg/kg) and xylazine (10 mg/kg). They were then inoculated intranasally (i.n.) with 20 μL of 1 × 10^3^ plaque-forming units (PFU)/animal of IAV diluted in PBS (0.1 LD^50^) or mock infected with PBS (Barbosa et al., 2014) and/or inoculated intratracheally (i.t.) with 30 μL of 1 × 10^4^ colony-forming units (CFU)/animal of Cg or PBS (controls) (Ferreira et al., 2015). We evaluated two sequential co-infection protocols: (i) inoculation of IAV 10, 7, or 3 days before infection (d.b.i.) with Cg; (ii) Infection with IAV, 3, 7, or 10 days after infection (d.p.i.) with Cg. Behavior, weight and survival of infected animals were monitored daily (Figure 1A). After this step, we selected the inoculation of IAV 3 d.b.i. with Cg (IAV+Cg) for all the next analysis.

In another set of experiments, mice (*n* = 6 animals per group) infected with IAV alone or IAV+Cg were treated daily with oral Oseltamivir, 31 mg/kg/day (Oselt, Tamiflu®, Roche). Alternatively, mice infected with Cg alone or IAV+Cg were treated daily with intraperitoneal fluconazole, 10 mg/kg/day (FLZ, Sigma-Aldrich, St. Louis, MO). Additional co-infected animals were treated with both FLZ and Oselt. Treatment with Oselt was started at the time of infection with IAV, whereas treatment with FLZ was started at the time of infection with Cg. In summary, experimental groups included: Not-treated (NT) Cg, IAV, and IAV+Cg; IAV treated with Oselt, Cg treated with FLZ, IAV+Cg treated with Oselt, IAV+Cg treated with FLZ, and IAV+Cg treated with Oselt+FLZ (Figure 1A). All the experiments were performed at least twice to confirm the data and the results were always reproducible.

### Behavioral analysis

Mouse behavior and function was assessed longitudinally in the groups: non-infected (NI), Cg, IAV and IAV+Cg (3 d.b.i.) using the SHIRPA protocol (Figure 1A) for neurological diseases (Santos et al., 2014). These tests provide reliable information on murine cerebral dysfunction and their general status. Individual parameters evaluated were grouped into five functional categories: neuropsychiatric state, motor behavior, autonomic function, muscle tone and strength, and reflex and sensory function. Individual parameters were summed up to determine a total score for each category (Santos et al., 2014).

### Fungal and viral burden and measurements of capsule size and zeta potential of *C. gattii*

After analysis of the survival curve, additional mice (*n* = 6 animals per group) were infected with Cg, IAV, or IAV+Cg or NI. The animals were anesthetized and euthanized by cervical dislocation 4 days post-infection with Cg (equivalent to 7 days infection by IAV). Lungs and brain were collected, homogenized in sterile PBS, plated onto SDA and incubated for 48 h at 37°C to determine the fungal burden, expressed as CFU per gram of tissue or milliliter of fluid (Costa et al., 2016). Likewise, viral loads in lung homogenates were assessed by titration in standard plaque assays on MDCK cells (Garcia et al., 2013). The viral titer was expressed as PFU per gram of tissue (Figure 1B).

In addition, bronchoalveolar lavage fluid (BAL) was aspirated as previously described (Santos et al., 2013). Fungal colonies recovered onto SDA after 48 h at 37°C from the BAL of Cg and IAV+Cg mice were further used for morphometric analyzes and Zeta potential (ζ) measurements (Figure 1B) as described previously (Nosanchuk et al., 1999; Ferreira et al., 2015). Briefly, Cg cells were visualized after suspension in Indian ink with an optical microscope (Axioplan; Carl Zeiss); subsequently the capsule size and total cell diameter size (diameter plus capsule) of at least 100 cells was measured in ImageJ 1.40g software (National Institutes of Health (NHI), Bethesda, MD). Capsule size was expressed in μm and the total cell diameter size was expressed as a frequency (%) of enlarged cells (>10 μm) compared to the size of typical cryptococcal cell (5–10 μm), according to Okagaki et al. (2010). The Zeta potentials of the yeast cells were calculated using a Zeta potential analyzer (Zetasizer NanoZS90; Malvern, United Kingdom).

### Histopathological analysis

Brain and lungs were collected (*n* = 6 animals per group), processed and stained with hematoxylin-eosin (HE) (Figure 1B). Then, the samples were blinded examined under light microscopy at 200x magnification. Each of the following parameters were graded as 0 (absent), 1 (mild), 2 (moderate), 3 (intense), and 4 (severe): lung tissue damage; degeneration of the airways (bronchioles); congestion and edema of the alveolar septa; alveolar edema; bronchiolar inflammatory infiltrate; and alveolar septa inflammatory infiltrate. Results were expressed and summarized as the average of the combined scores for the distinct parameters evaluated.

### Lung myeloperoxidase (MPO) and N-acetylglucosaminidase (NAG) activities and cytokine and chemokine levels

For the better understanding of the increased susceptibility caused by IAV 3 d.b.i., six mice per group were infected with IAV or mock infected (NI), and were euthanized 3 days after infection (Figure 1C). MPO and NAG assays were performed on lung pieces (100 mg) (Costa et al., 2016), providing an indirect measurement of neutrophil and macrophage accumulation in lungs, respectively. The concentrations of IL-1β, IL-4, IL-6, IL-10, IFN-γ, TNF-α, and CXCL1 were measured by ELISA using commercially available antibodies from DuoSet Kits (R&D Systems, Minneapolis, MN) according to the manufacturer's instructions. Fragments of lung tissue (100 mg) were homogenized with 1 ml of extraction buffer, prepared with phosphate buffered saline—PBS (pH 7.4) containing anti-proteases (0.1 mM phenylmethilsulfonyl fluoride, 0.1 mM benzethonium chloride, 10 mM EDTA and 20 KI aprotinin A, all purchased from Sigma-Aldrich) and 0.05% Tween 20. The expression of IFN-α4 and IFN-β genes was measured in lung tissue by qRT-PCR, using primers specific for murine samples (Costa et al., 2014). The primers used were: mIFNα4, 5′-CCA CAG CCC AGA GAG TGA CCA GC-3′ (forward) and 5′-AGG CCC TCT TGT TCC CGA GGT TA-3′ (reverse); mIFNβ, 5′-GAA AGG ACG AAC ATT CGG AAA T-3′ (forward) and 5′-CGT CAT CTC CAT AGG GAT CTT GA-3′ (reverse); and 18S ribosomal RNA, 5′-CGT TCC ACC AAC TAA GAA CG-3′ (forward) and 5′-CTC AAC ACG GGA AAC CTC AC-3′ (reverse).

### Phagocytosis, intracellular proliferation, ROS, peroxynitrite, and nitric oxide production by macrophages

Initially, phagocytosis was studied *in vivo* during co-infection by counting yeast cells internalized by phagocyte cells in BAL (Figure 1B). The phagocytic index was determined as the number of internalized yeast cells per 100 mononuclear cells and results were expressed as a percentage. Further, bone marrow-derived macrophages (BMDM) were isolated as described previously (Ribeiro et al., 2017). Briefly, bone marrow cells recovered from mice femurs and tibias were counted using a hemocytometer and the concentration adjusted to 2 × 10^6^ cells/mL for incubation in BMM medium (RPMI supplemented with 30% L929 growth conditioning media, 20% bovine fetal serum [Gibco], 2 mM glutamine [Sigma-Aldrich], 25 mM HEPES pH 7.2, 100 units/mL of penicillin-streptomycin [Life Technologies]). Fresh media were added every 48 h. BMDMs were collected on day 7 and used for subsequent experiments. Then, 2 × 10^5^ BMDM/mL were plated into 24-well plates for determining phagocytosis and Intracellular Proliferation Rate (IPR) and 96-well plates for detection of Reactive Oxygen Species (ROS) and peroxynitrite (PRN), and incubated overnight in RPMI supplemented with 10% fetal bovine serum at 37°C under 5% CO_2_ (Figure 1D).

To this aim, cells were inoculated with IAV (M.O.I. = 1) 2 h before (IAV+Cg) infection with Cg (5 cells:1 yeast) or with Cg or IAV alone (Figure 1D). The phagocytic index post 3 h inoculation with Cg was determined as described above. For the IPR assay, cell culture supernatants were removed and non-internalized and adherent yeast cells were removed by two washes with PBS. BMDMs then were lysed with 200 μL of sterile distilled water for 30 min at 37°C, and 50 μL of this suspension were plated on SDA for CFU determination. The IPR was calculated as the quotient of the CFU/mL at 24 and 3 h post Cg inoculation (Ma et al., 2009; Ribeiro et al., 2017).

The above experiments were also performed using RPMI-1640 without phenol red (Sigma-Aldrich), followed by incubation with 2′,7′-dichlorofluoresceindiacetate (DCFH-DA; Invitrogen, Life Technologies, Carlsbad, CA, USA) or dihydrorhodamine-123 (DHR-123; Invitrogen) for ROS and PRN measurements, respectively. Fluorescence was assessed 3 h post-inoculation of Cg using a fluorometer (Synergy 2 SL Luminescence Microplate Reader; Biotek) with excitation and emission wavelengths of 485/530 nm. The data are expressed as arbitrary units of fluorescence (Ferreira et al., 2013). The supernatant from the phagocytosis assay was used to quantify nitric oxide (NO) production using the Griess assay. The nitrite concentration was determined by extrapolation from a sodium nitrite standard curve, read at 540 nm with a microplate reader. For all these tests, we also have groups pre-incubated overnight with IFN-γ (50 U/mL).

### Statistical analyzes

All statistical analyzes were performed using GraphPad Prism, version 5.00, for Windows (GraphPad Software, San Diego, CA, USA) with *p* < 0.05 considered to be significant. Kaplan-Meier survival curves were generated and analyzed using the log rank test. SHIRPA data were analyzed using the area under the curve, followed by analysis of variance (ANOVA) and a Tukey test. The histopathology analysis, phagocytosis and IPR assay, ROS, PRN, and NO measurements were analyzed by ANOVA followed by Tukey test to compare different groups. The results of CFU/g, PFU/g, capsule size, zeta potential, MPO and NAG activities, cytokines and chemokines levels were analyzed by the non-parametric Friedman test, used to compare two groups. All the tests were performed at least twice and the results were always reproducible.

## Results

### Co-infection with IAV and *C. gattii* leads to increased morbidity and reduces survival rates in mice

**Figure 2:**
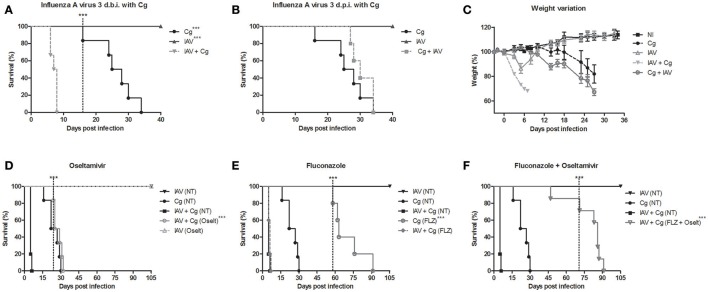
Survival curve (**A,B**), weight variation **(C)** and treatment with antiviral oseltamivir **(D)**, antifungal fluconazole **(E)**, and combined treatment **(F)** of *C. gattii* co-infection with Influenza A virus H1N1 (PR8). Six mice per group were infected i.t. with 1 × 10^4^ CFU of *C. gattii*, or a sublethal dose of Influenza A virus i.n. (1 × 10^3^ PFU/animal) or both. Cg: *C. gattii*; IAV: Influenza A virus; IAV+Cg: Influenza A virus 3 days before infection (3 d.b.i.) to *C. gattii*; Cg+IAV: Influenza A virus 3 days post-infection (3 d.p.i.) to *C. gattii*. NI: group not infected; NT, Non-treated group; FLZ, Fluconazole; Oselt, Oseltamivir. - - - -The dotted vertical line indicates when the statistically difference between the groups began. ^***^p < 0.0005. Data are representative of three independent experiments.

Initially we demonstrate that a sublethal dose of influenza virus alters the survival of mice infected with *C. gattii*. Survival studies reveal that infection with Cg alone causes 100% mortality by 35 days of infection (Figures 2A,B). Mice inoculated with IAV alone, lost substantial weight up to day 6 (Figure 2C), but survived the infection (Figure 2A). Notably, infection with IAV 3 days before Cg resulted in faster and higher weight lost and reduced survival of infected mice (death within 8 days) (*p* < 0.05) compared with mice infected with Cg (Figures 2A,C). Identical results were obtained using *C. gattii* R265 and *C. neoformans* H99 strains (Supplementary Figure 1). In contrast, when we compare Figures 2A,B, we observed that mice infected with IAV 3 days after Cg did not present difference in survival compared to animals infected only by Cg (Figure 2B); otherwise mice infected with IAV 3 days before Cg has a marked increase in the rate of mortality. In the same way, there was no difference in survival of mice infected with IAV 10 or 7 days prior to inoculation with Cg (Supplementary Figure 2). Similarly, there was no difference in survival of mice when IAV was inoculated 7 or 10 days after Cg (Supplementary Figure 2).

Our previous study showed that the IAV load in lungs peaked at about the fourth day after infection and that IAV was cleared by day 10 (Barbosa et al., 2014). Therefore, our data strongly suggested a link between high viral replication and increased susceptibility to Cg during acute influenza infections. To test this hypothesis, we treated mice daily with oseltamivir and/or fluconazole. Remarkably, we verified that treatment with oseltamivir delayed the mortality in co-infected mice (Figure 2D). While treatment with the antifungal fluconazole improved survival of mice infected with Cg, it did not improve survival in co-infected mice (Figure 2E). However, combined treatment (Oselt+FLZ) significantly improved survival in co-infected mice (Figure 2F).

**Figure 3:**
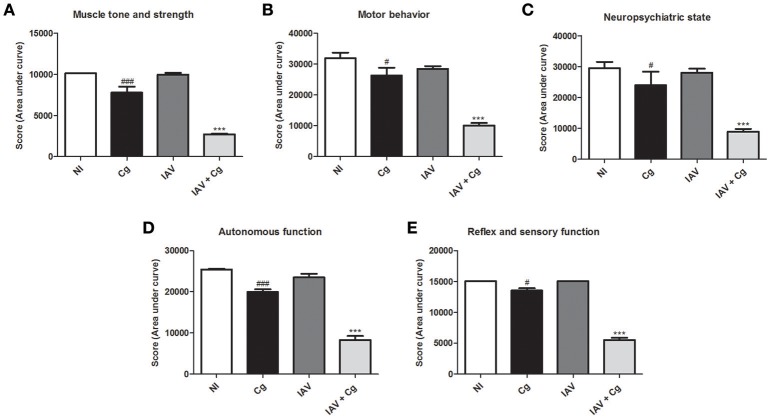
Behavioral assessment of mice. The behavior performance was assessed daily by the SHIRPA protocol and the results are divided in five functional categories: muscle tone and strength **(A)**; motor behavior **(B)**; neuropsychiatric state **(C)**; autonomous function **(D)**; reflex and sensory function **(E)**. Five animals per group were submitted to the SHIRPA Protocol. NI, group not infected; Cg, *C. gattii*; IAV, Influenza A virus; IAV+Cg, Influenza A virus 3 days before infection (3 d.b.i.) to *C. gattii*. ^#^*p* < 0.05, ^###^*p* < 0.0005 (statistically significant difference when compared to NI); ^***^p < 0.0005 (statistically significant difference when compared to NI, Cg, and IAV). Data are representative of three independent experiments.

Application of the SHIRPA protocol showed that infection with IAV resulted in undetectable cerebral impairment while infection with Cg did cause cerebral dysfunction. Moreover, morbidity in co-infected mice was increased compared with all other groups, showing a severe decline (*p* < 0.0005) in each of the five functional categories evaluated (Figures 3A–E).

### Co-infection (IAV+Cg) increases fungal burden in brain and causes severe histopathological changes in lungs

**Figure 4:**
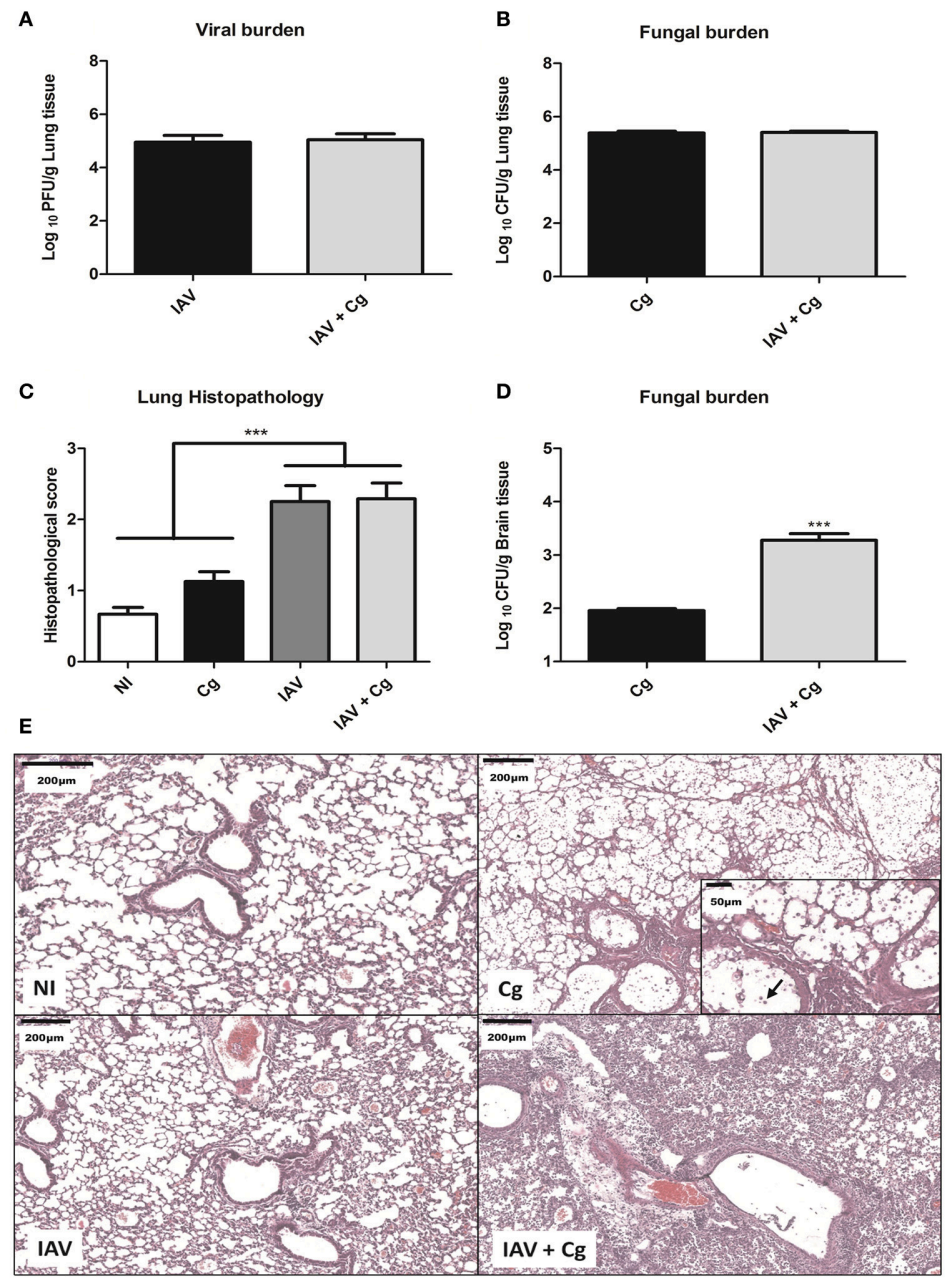
Viral and fungal burdens and histopathology. Six mice per group were inoculated i.t. with 1 × 10^4^ CFU of *C. gattii*, or i.n. 1 × 10^3^ PFU/animal of Influenza A virus or both. After 4 days of infection with Cg (equivalent to 7 days infection by IAV) the animals were ethically euthanized to assess the viral and fungal burden and histopathology. Histological sections of lungs were stained with H&E and visualized at 200 × magnification and performed at least 10 fields per coverslip. Viral burden in the lungs **(A)**. Fungal burden in the lungs **(B)**. Histopathological score of the lungs **(C)**. Fungal burden in the brain **(D)**. Histopathological panel: representative pictures of the histopathology of the lungs (Scale bar = 200 μm) **(E)**. The arrow indicates cryptococci in the amplified box (Scale bar = 50 μm). Cg, *C. gattii*; IAV, Influenza A virus; IAV+Cg, Influenza A virus 3 days before infection (3 d.b.i.) with *C. gattii*. NI, non-infected group. ^***^p < 0.0005 (statistically significant difference when compared to Cg). Data are representative of two independent experiments.

To evaluate the disease caused by Influenza-*Cryptococcus* co-infection, mice were infected with IAV 3 days before infection by Cg (IAV+Cg) or infected only by Cg; IAV or non-infected (NI). Four days (7 days of protocol) post-infection with Cg, these animals were ethically euthanized for disease profile investigation. We found no significant differences (*p* > 0.05) in the viral (Figure 4A) and fungal burdens (Figure 4B) in lungs of mice infected with IAV or Cg alone, respectively, compared with co-infected mice. However, fungal loads in the brain were significantly higher in co-infected mice than in mice infected with Cg alone (Figure 4D). In addition, histopathological scores were higher in lungs of mice infected with IAV or co-infected when compared with those infected with Cg alone (Figure 4C). Histopathological findings in the groups infected with IAV alone or co-infected showed that the virus directly damages the lung structure, including the mild airway degeneration, congestion and edema of the alveolar septa and increased bronchial and septal inflammation (Figure 4E). The presence of the fungus is evidenced by arrow in the Cg group (amplified box, Figure 4E).

### Co-infection increased capsule thickness and electronegativity of the fungal cell surface

**Figure 5:**
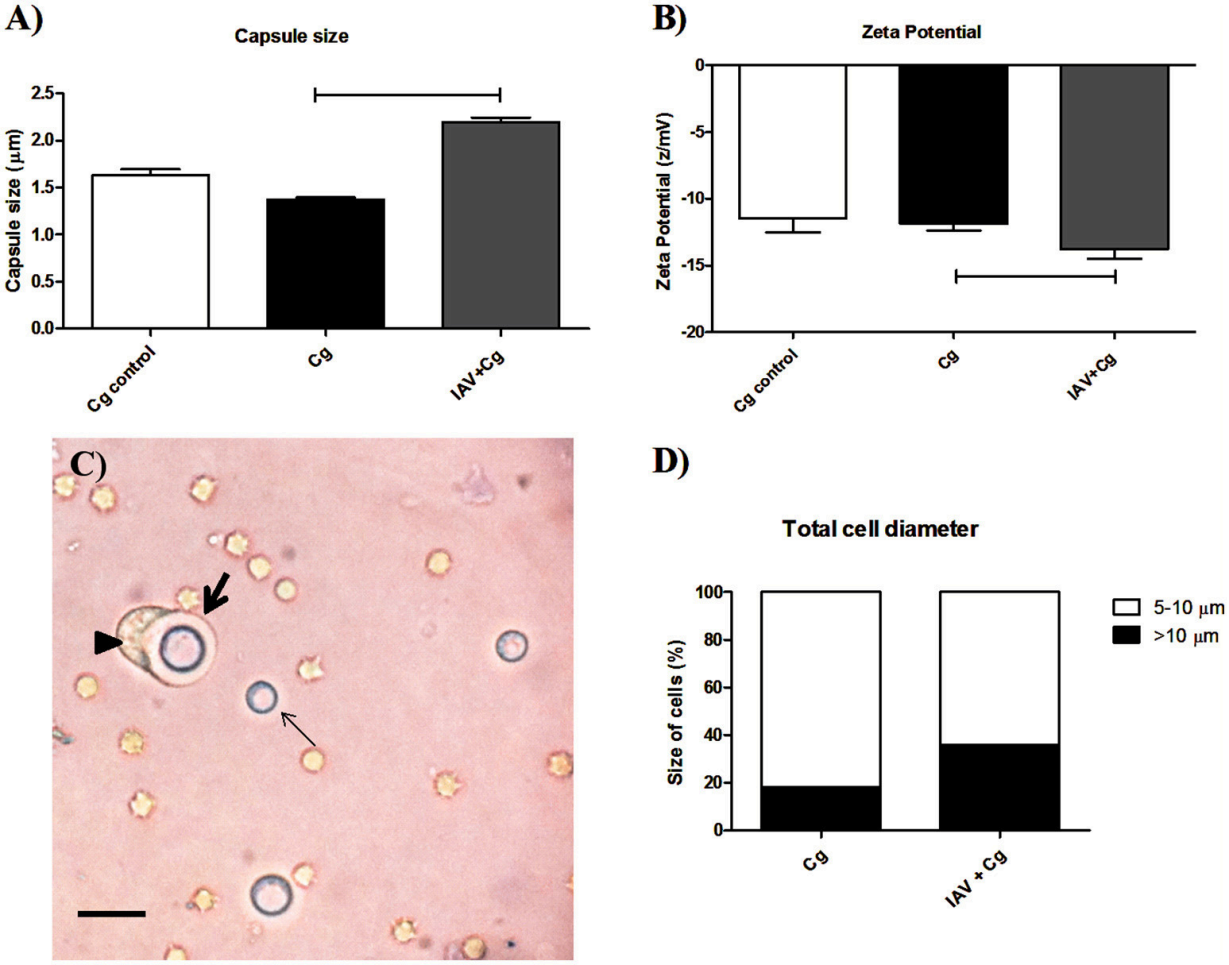
Morphometric assay of cryptococci from bronchoalveolar lavage fluid (BAL). Six mice per group were inoculated i.t. with 1 × 10^4^ CFU of Cg, or i.n. 1 × 10^3^ PFU of IAV or both (IAV+Cg); 4 days after infection by Cg the animals were ethically euthanized and bronchoalveolar lavage fluid (BAL) aspirated. Colonies of Cg recovered from BAL after 48 h incubation at 37°C in Sabouraud dextrose agar (SDA) were further submitted to morphometric analysis. Capsule size (μm) **(A)**. Zeta potential **(B)**. Picture obtained from the BAL of a co-infected mouse **(C)**. Total cell diameter size **(D)** expressed as the percentage of cells with 5–10 μm (size of typical cryptococcal cells) or with >10 μm (enlarged cells). The arrowhead shows a phagocytic cell attempting to engulf an enlarged encapsulated *Cryptococcus* cell (thick arrow), and the thin arrow shows a non-enlarged *Cryptococcus* cell. Scale bar = 20 μm. Cg control, fungi maintained *in vitro*; Cg, fungal recovered from mice infected with Cg; IAV+Cg, fungal recovered from IAV-infected mice 3 d.b.i. with *C. gattii*. The statistically significant differences among the groups are represented by the horizontal lines (*p* < 0.05). Data are representative of two independent experiments with six animals per group in triplicate assays.

Considering the higher transmigration of Cg to CNS, we verified if this fact could be associated with the fungal adaptation (morphological modifications of Cg during co-infection) to the host in the presence of virus. For this, colonies recovered from BAL of mice co-infected or infected only by Cg were submitted to capsule size and zeta potential determination. Our data demonstrated an increased capsule size (Figure 5A) and increased electronegativity (Figure 5B) in the fungal cells recovered from BAL of mice infected with IAV+Cg, when compared with those infected with Cg alone and cultured fungi (Cg control). Figure 5C demonstrates a phagocyte (arrow head) attempting to engulf an enlarged encapsulated Cg cell (thick arrow, Figure 5C). In the same way, our results for the total cell diameter size showed the higher proportion of enlarged cells (>10 μm) in the co-infected group (35.9%), while mice infected only by Cg present only 18.0% of enlarged cells (Figure 5D).

### IAV increased expression of IFN-α4 and IFN-β and pro-inflammatory mediators, but decreased IFN-γ levels

**Figure 6:**
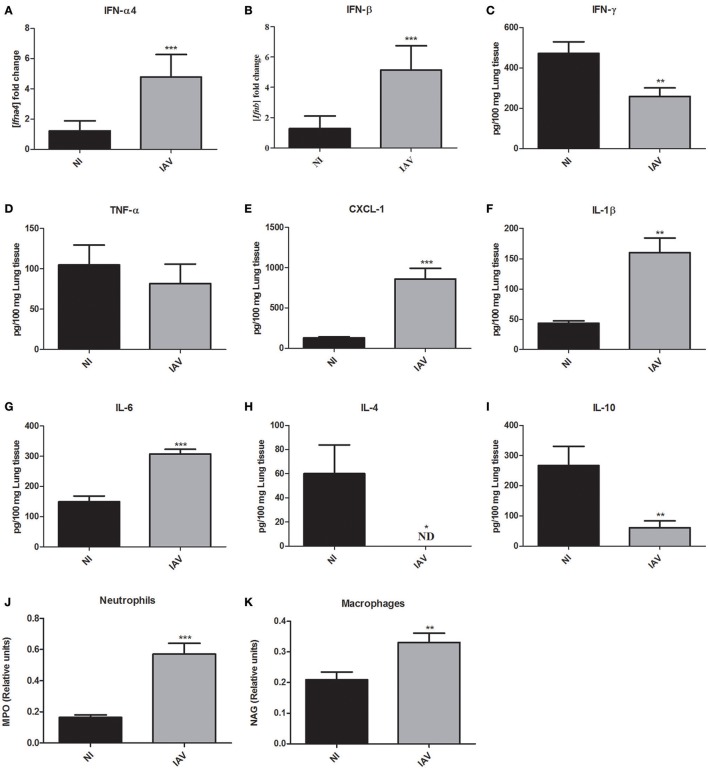
Measurement of cytokines and chemokine in the lungs and myeloperoxidase (MPO) and N-acetylglucosaminidase (NAG) activities in the lungs of mice after 3 days of influenza A virus (IAV) infection compared to non-infected (NI) group. Six mice per group were infected only with IAV or non-infected (NI) to investigate the immunological response induced by influenza. The animals were ethically euthanized 3 days after IAV infection to assess cytokines and chemokine levels and MPO and NAG activities in the lungs. Fold increase of mRNA of IFN-α4 **(A**) and IFN-β **(B)**. Levels of IFN-γ **(C)**, TNF-α **(D)**, CXCL-1 **(E)**, IL-1β **(F)**, IL-6 **(G)**, IL-4 **(H)**, and IL-10 **(I)**. The relative number of neutrophils was indirectly measured by the MPO activity **(J)** and the relative number macrophages was indirectly measured by the NAG activity **(K)**. ^*^p < 0.05, ^**^p < 0.005, and ^***^p < 0.0005 (statistically significant difference when compared to NI). ND, non detected. Data are representative of three independent experiments with six animals per group.

Given the severe histopathological alterations caused by IAV infection, we investigated the host response against IAV that may also be responsible for the increased susceptibility to secondary *C. gattii* infection. Further, to determine the inflammatory profile of mice at the time they would be infected with Cg, mice were infected only by IAV (1 × 10^3^ PFU) or NI and they were ethically euthanized and lungs collected 3 days after IAV infection. The expression of the genes of type 1 interferons, i.e., interferon alpha (IFN-α4) and beta (IFN-β) were significantly increased (*p* < 0.0005) in the IAV infection (Figures 6A,B, respectively). Interestingly, the levels of interferon gamma (IFN-γ) were reduced (*p* < 0.005) in comparison with non-infected mice (Figure 6C). Additionally, the levels of tumor necrosis factor-alpha (TNF-α) were not altered by IAV (Figure 6D). We verified increased levels of CXCL-1 (Figure 6E), interleukin 1-β (IL-1β) (Figure 6F), and IL-6 (Figure 6G). Moreover, the levels of cytokines IL-4 (Figure 6H) and IL-10 (Figure 6I) were lower than those in NI mice. Here we used MPO as an indirect measurement of neutrophil accumulation, since MPO is the most abundant pro-inflammatory enzyme stored in the azurophilic granules of neutrophilic granulocytes (Pulli et al., 2013). In the same way, an estimate of macrophage infiltration is given by the level of NAG which is present in high specific activity in activated macrophages (Bailey, 1988). The activities of the enzymes MPO (Figure 6J) and NAG (Figure 6K) were significantly (*p* < 0.05) increased in lungs of mice infected with IAV compared with NI mice, suggesting that neutrophils and macrophages, respectively, had been recruited to the site of infection.

### Co-infection impairs phagocytosis of *C. gattii* and production of ROS and increases the intracellular proliferation rate

**Figure 7:**
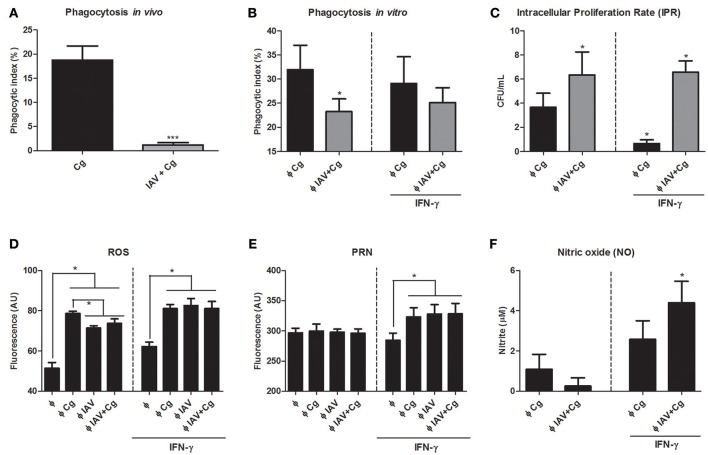
Assessment of phagocytosis and intracellular proliferation of Cg in murine BMDM. The phagocytosis assay was performed *in vivo* by counting internalized yeast cells in bronchoalveolar lavage fluid (BAL) cells aspired from mice (*n* = 6 animals per group) infected with IAV+Cg or with only Cg; and *in vitro* with bone marrow-derived macrophages (BMDM). *In vivo* phagocytosis **(A)**, *in vitro* phagocytosis after 3 h of BMDM infection with Cg **(B)**, intracellular proliferation rate (IPR) after BMDM infection with Cg **(C)**. Production of ROS after 3 h of BMDM infection with Cg **(D)**, Peroxynitrite after 3 h of BMDM infection with Cg **(E)** and nitric oxide (NO) after 3 h of BMDMs infection with Cg **(F)**. For *in vitro* phagocytosis, assessment of IPR and ROS, PRN, and NO production, independent groups of BMDM were infected with Cg, IAV, or IAV 2 h before infection with Cg (IAV+Cg) and left unstimulated or stimulated with IFN-γ. Φ: BMDM. ^*^p < 0.05; ^***^p < 0.0005 (statistically significant difference when compared to Cg). Data are representative of three independent experiments consisting of six replicates.

In order to assess the influence of IAV infection in Cg phagocytosis, we performed the *in vivo* and *in vitro* phagocytosis assays. The phagocytic index of Cg, as assessed using mononuclear cells of BAL aspirated from IAV+Cg-co-infected mice, was reduced in comparison with those infected with Cg alone (Figure 7A). These data were confirmed in the *in vitro* assay using BMDM (Figure 7B). Furthermore, the IPR assay revealed an increased ability of Cg to grow inside BMDMs that had been virus-infected 2 h prior to the addition of Cg (IAV+Cg) (Figure 7C). In addition, challenge with IAV or IAV+Cg reduced the ability of macrophages to produce ROS compared to cells infected with Cg (Figure 7D), but not PRN (Figure 7E) and NO (Figure 7F). Treatment with IFN-γ improved the oxidative burst activity of macrophages and decreased the IPR for BMDM challenged with Cg alone. However, in co-infected BMDMs, IAV impaired the fungicide activity of macrophages independently of the increase in ROS, PRN, and NO production induced by IFN-γ.

## Discussion

IAV is a major cause of epidemics and pandemics in humans, occasioning high morbidity and mortality worldwide (Saxena et al., 2017). The IAV infections are widely associated with secondary invasive aspergillosis, even among immunocompetent hosts, including those without classic risk factors (Kwon et al., 2013; Alshabani et al., 2015; Crum-Cianflone, 2016). Although the contribution of secondary bacterial infection to the morbidity and mortality of influenza has been studied extensively, there is a remarkable paucity of information on co-infection with other respiratory pathogens (Peltola and McCullers, 2004; Smith et al., 2013; Duvigneau et al., 2016). *Cryptococcus gattii* is an airborne fungal pathogen, which infects the lungs and secondarily, the CNS. Unlike the majority of infections with *C. neoformans*, those caused by *C. gattii* frequently occur in non-immuncompromised hosts (Bielska and May, 2016). As far as we know, there are only two case reports describing an association between *C. neoformans* and influenza virus (Hosseinnezhad and Rapose, 2012; Gupta et al., 2015), both caused by the 2009-pandemic H1N1 influenza A, showing clinical evidence of co-infection among these pathogens. Because Cg is an emergent and still poorly understood pathogen, which infects apparently healthy hosts and displays the lung tropism as widespread IAV, we evaluated the impact of co-infection in a murine model.

Our preliminary findings showed that a deleterious effect of co-infection was evident only in mice exposed to IAV 3 days before inoculation with Cg. Notably, day 3 of IAV infection coincides with high levels of virus in infected lungs (Ayala et al., 2011; Pan et al., 2013; Barbosa et al., 2014). The importance of active viral multiplication in this predisposition to severe pulmonary cryptococcal disease was demonstrated by treatment with the antiviral oseltamivir, which delayed mortality of mice co-infected. Similar sequence of effects were reported in mice initially infected with IAV and inoculated later with *Legionella pneumophila*, which displayed increased weight loss and succumbed to infection, whereas the animals infected only with one pathogen survived (Jamieson et al., 2013).

Although there were no differences in viral or fungal loads in lungs of mice co-infected when compared with those of mice infected with IAV or Cg alone, cerebral loads of cryptococci were higher in the presence of co-infection. These results may explain their impaired neuronal responses as assessed by SHIRPA and the worse prognosis. Moreover, infection with IAV alone or with Cg resulted in a more intense inflammatory infiltrate and severe lung damage. Indeed, the influenza virus itself is known to impair normal ciliary function (Tavares et al., 2017). In general, vulnerability to secondary infections is associated with lung damage, impairment of host defense mechanisms and impairment of phagocytosis (Smith et al., 2013; Cauley and Vella, 2015; Tavares et al., 2017). We suggest that (i) IAV-associated lung injury along with (ii) impairment of phagocytosis of the yeast and (iii) an increased inflammatory response observed in the lungs of mice may enhance the dissemination of *C. gattii* to the CNS, leading to the poor prognosis of the disease, similar to the previously demonstrated in cases of bacterial co-infections (Pittet et al., 2010; Kash et al., 2011; Liu et al., 2013).

In order to better understand the host response to IAV, we demonstrated that an increased proinflammatory cytokine profile correlated with recruitment of inflammatory cell populations. In particular, we noted higher levels of cytokines IL-1β, IL-6, and chemokine CXCL-1 and decreased levels of IL-4 and IL-10 in the presence of IAV, but no change in TNF-α production. Together with these data, the increased expression of IFN-α4 and IFN-β that we verified in the presence of IAV reflects the development of the antiviral state, which is known to protect the cells from the virus (Randall and Goodbourn, 2008; Tavares et al., 2017). Previous studies have shown that IAV infection can induce the production of type 1 IFN, suppress the macrophage activity and disrupt the normal ciliary clearance, which lead to an increased susceptibility to different pathogens, including the fungus *Aspergillus* spp. (Crum-Cianflone, 2016) and secondary bacterial infections (Shahangian et al., 2009; Kudva et al., 2011; Li et al., 2012).

Intriguingly, 3 days after infection, we observed a significant decrease in IFN-γ levels in mice infected with IAV. These data corroborate the study of Nguyen et al. (2000), who demonstrated that type 1 interferons (IFN-α/β) inhibit the production of IFN-γ by NK and T cells by a process that requires STAT1 (signal transducer and activator of transcription 1) regulation (Nguyen et al., 2000). Accordingly, Arimori et al. (2013) verified that knockout mice for the receptor of type 1 interferon (IFNAR KO) infected by influenza PR8 produce increased amounts of IFN-γ in the lungs (Arimori et al., 2013). Moreover, Sato et al. (2015) demonstrated the increased clearance of *C. neoformans* in mice knockout for the receptor of IFN-α (IFNAR1KO), which exhibited enhanced synthesis of IFN-γ and the IL-4. The authors also suggested that type I IFNs may be involved in the negative regulation of early host defense to this infection (Sato et al., 2015). Taken together, these data support our finding that IFN-γ is reduced when type 1 interferon is increased.

The role of IFN-γ in fungal infections has been demonstrated exhaustively and it is considered to be a key cytokine in anti-cryptococcal host defense (Hardison et al., 2010; Davis et al., 2015). Classical studies showed that IFN-γ promotes macrophage engulfment and killing of the fungus (Flesch et al., 1989; Kawakami et al., 1995; Hardison et al., 2010; Davis et al., 2015). In addition, M1 (classically-activated) macrophages are considered efficient killers via oxidative bursts, which are usually activated by IFN-γ (Hardison et al., 2010; Davis et al., 2015). Also, Panackal et al. (2015), in a study with non-HIV related cryptococcal disease, proposed that macrophage activation defects along with intact T-cell activation is pivotal to cause increased susceptibility of the patient to the fungus and severe CNS disease (Panackal et al., 2015). These findings support our observation that IAV+Cg led to reduced macrophage fungicidal activity, i.e., reduced ability to engulf Cg, lower ROS production and increased fungal proliferation, which is correlated with the severity of cryptococcosis disease.

Surprisingly, IFN-γ exposure did not control intracellular proliferation rates during co-infection although it did augment macrophage oxidative bursts. Uetani et al. (2008) demonstrated that IAV subverts the response mediated by IFN-γ through effects on the intracellular signaling pathways (Uetani et al., 2008). The reduced phagocytosis during co-infection may also be a consequence of the morphological plasticity of *C. gattii*, which has the ability to enlarge its polysaccharide capsule, neutralize ROS and peroxynitrite (Frases et al., 2009; Kronstad et al., 2011; Chen et al., 2014; Urai et al., 2015). In the same way, the higher frequency of enlarged cells during co-infection may be related to the increased virulence of *Cryptococcus* spp. through of decreasing phagocytosis, leading to the fungal persistence in the host and enhanced dissemination to the CNS (Okagaki et al., 2010; Zaragoza and Nielsen, 2013). In addition, the capsule became more electronegative, which may inhibit the interaction between Cg and macrophages (Nosanchuk et al., 1999). We speculate that Cg uses the macrophage, since its fungicidal activity is reduced, to evade the inflammatory response and enters the CNS by the well-established “Trojan horse” mechanism (Sorrell et al., 2016; Santiago-Tirado et al., 2017), leading to the poor prognosis of the disease.

In conclusion, we have shown that IAV (at the peak of infection) causes (i) lung tissue damage, (ii) impairment of phagocytosis, and (iii) an immune profile that likely triggers *C. gattii* disease of increased severity. Thus, it may be considered as a predisposing factor for this mycosis. In addition, we provide unprecedented knowledge on the interaction between these infectious agents and the host during co-infection.

## Author contributions

LO: performed all experiments procedures involving animals analysis, immunological assays, flow cytometry, experimental procedures *in vitro*, results analysis and wrote the paper. MC: performed experimental procedures *in vivo, in vitro*, as well as theoretical aspects of the paper. TM, RB, HC, and NR: contributed in all experimental procedures with animals. PS: contributed in experiments procedures of immunological assays. GF: conducted the morphometric analysis and Zeta potential of Cg. LR: contributed in immunological assays. AG: carried out experiments with influenza virus, such as viral stock preparation and viral titration. CF: participated in the analysis of inflammatory factors and theoretical aspects of immunology of the paper. MP: performed the histopathological analysis. JD, and TS: substantial contributions to design some experiments, important intellectual content for research development and theoretical aspects of the paper, revising it critically. DS, and AM: supervised the development of tests in their expertise area and discussed the results. DS: coordinated the project, designed and supervised the study, and wrote the paper.

### Conflict of interest statement

The authors declare that the research was conducted in the absence of any commercial or financial relationships that could be construed as a potential conflict of interest.
