# Supplementary material for: Influenza A Virus as a Predisposing Factor for Cryptococcosis
Source: Front Cell Infect Microbiol. 2017 Sep 26;7:419. doi: 10.3389/fcimb.2017.00419 (PMC5622999; doi:10.3389/fcimb.2017.00419)
Supplement: Supplementary file 1 [file DataSheet1.doc]

Supplementary Material

# Influenza A virus as a predisposing factor for cryptococcosis

Lorena Vívien Neves de Oliveira, Marliete Carvalho Costa, Thaís Furtado Ferreira Magalhães, Rafael Wesley Bastos, Patrícia Campi Santos, Hellem Cristina Silva Carneiro, Noelly de Queiroz Ribeiro, Gabriella Freitas Ferreira, Lucas Secchim Ribeiro, Ana Paula de Faria Gonçalves, Caio Tavares Fagundes, Marcelo Antônio Pascoal-Xavier, Julianne T. Djordjevic, Tania C. Sorrell, Daniele Glória de Souza, Alexandre Magalhães Vieira Machado, Daniel Assis Santos*

***Correspondence:** Daniel A. Santos, [das@ufmg.br](mailto:das@ufmg.br) or dasufmg@gmail.com

## Supplementary Figures

**Supplementary Figure 1 – Survival curves of mice infected or coinfected with IAV and different strains of *Cryptococcus*.** Other *Cryptococcus* strains were tested for coinfection with Influenza A PR8 H1N1 virus (IAV) in murine model (n = 6 animals per group), as described in Methods. The strains tested were *C. gattii* (Cg) R265, VGII **(A)** and *C. neoformans* (Cn) H99, VNI **(B)**, both with an inoculum of 1x10^4^ UFC/animal. The survival of mice coinfected (IAV 3 days before *Cryptococcus* infection) significantly decreased for strains R265 and H99 tested. The dotted vertical line indicates when the most noticeable difference between the groups began. **(*p* < 0.05); ***(*p* < 0.005).

**Supplementary** **Figure 2 – Survival curves of mice infected with *C. gattii* (Cg) alone, Influenza A virus (IAV) alone or coinfected with IAV and Cg.** We tested different times of mice infection (n = 6 animals per group) with IAV in relation to Cg infection. **(A)** Coinfection with IAV 10 days before infection (10 d.b.i.) with Cg (IAV+Cg); **(B)** Coinfection with IAV 7 days before infection (7 d.b.i.) with Cg (IAV+Cg); **(C)** Coinfection with IAV 7 days post-infection (7 d.p.i.) with Cg (Cg+IAV); **(D)** Coinfection with IAV 10 days post-infection (10 d.p.i.) with Cg (Cg+IAV).
